# Self-assembled monolayers for electrostatic electrocatalysis and enhanced electrode stability in thermogalvanic cells†

**DOI:** 10.1039/d3sc06766a

**Published:** 2024-04-03

**Authors:** Kristine Laws, Mark A. Buckingham, Leigh Aldous

**Affiliations:** a Department of Chemistry, Britannia House, King's College London London SE1 1DB UK leigh.aldous@kcl.ac.uk

## Abstract

Waste heat is ubiquitous; as such, sustainable and long-lasting devices are required to convert it into more useful forms of energy that can make use of this abundant potential resource. Thermogalvanic cells (or thermocells) can use the thermoelectrochemical properties of redox couples to achieve this; entropy-driven redox reactions allow them to act as liquid thermoelectrics. However, excellent electrocatalysis at the electrode surface is required for optimum conversion efficiency. Serendipitous observation of Nafion-based electrocatalysis prompted the exploration of electrostatically charged self-assembled monolayers (SAMs) inside a thermocell. Both electrostatic electrocatalysis and improved electrode stability were observed; in an aqueous K_3_[Fe(CN)_6_]/K_4_[Fe(CN)_6_]-based cell, modification with (3-trimethylammonium bromide)thiopropane resulted in higher electrical power, and protection against [Fe(CN)_6_]^3−/4−^-induced gold passivation, relative to bare gold. Molecular-based electrostatic electrocatalysis could be an alternative to precious metal-based nanomaterial electrocatalysis, and could be integrated with (nano)carbon-based electrodes to further enhance the ability of thermogalvanic and other electrochemical energy conversion devices, *e.g.* redox flow batteries.

## Abstract 2

Thermogalvanic cells can convert temperature gradients (*e.g.* waste heat) into electricity; here we demonstrate how electrostatically charged self-assembled monolayers can act as molecular electrocatalysts, plus reduce electrode passivation.

## Graphical abstract

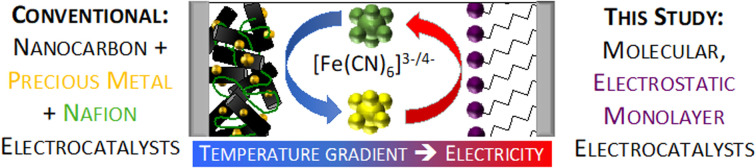

## Introduction

Sustainability and ‘Net Zero’ objectives necessitate improved efficiency and accessing new renewable energy sources. Approximately two-thirds of primary energy is lost as low-grade waste heat, while biological processes and the sun provide additional gigantic quantities of untapped low grade waste heat, making these valuable potential contributors.^1^ Numerous recent electrochemical studies have focussed upon harvesting low-grade waste heat (below 100 °C), because no efficient technology exists in this temperature region that is commercially viable.^1,2^ One enticing prospect is thermogalvanic electrochemical cells (or thermocells). These relatively simple devices comprise two electrodes and a typically aqueous redox active electrolyte, and thus have promising green and sustainable credentials.^3,4^

**Fig. 1:**
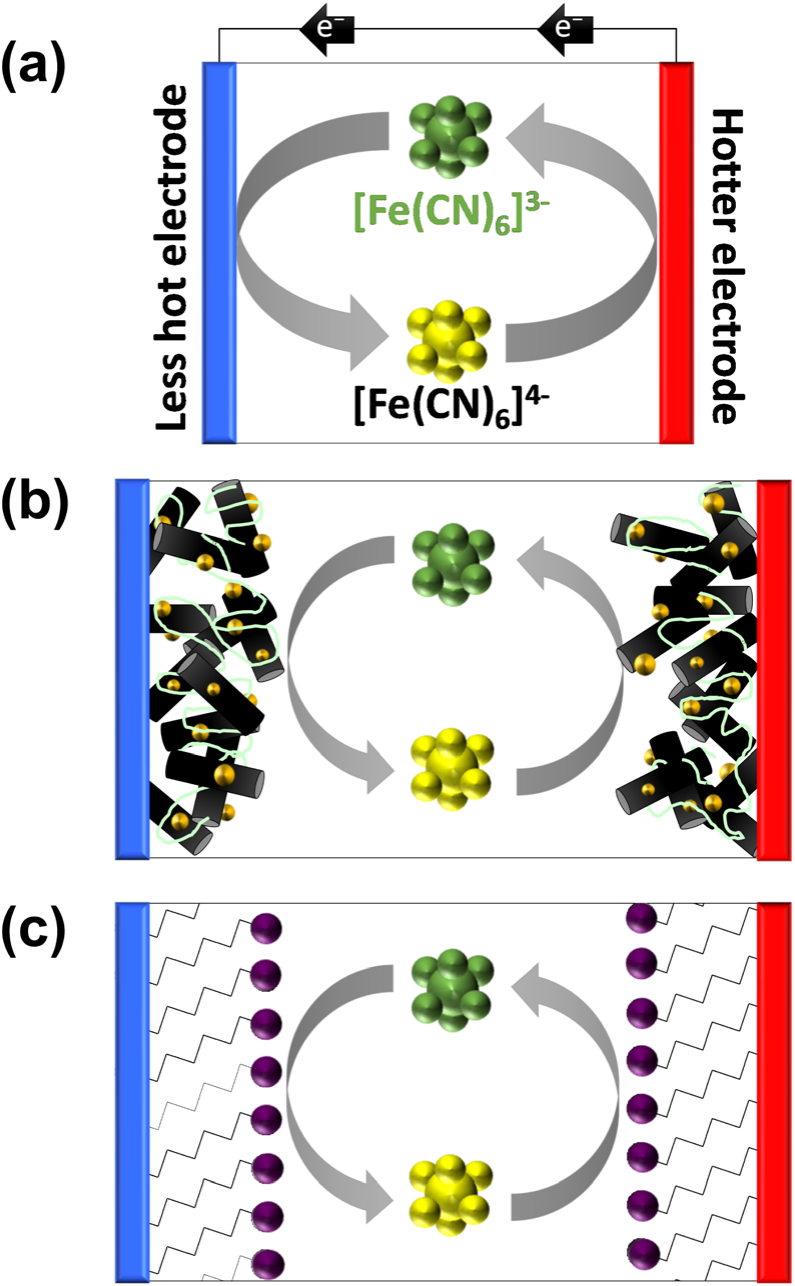
Schematic showing (a) the basic operation of a [Fe(CN)_6_]^3−/4−^ thermogalvanic cell (thermocell), converting a temperature gradient into a flow of electrical current, and (b) thermocell containing a hypothetical carbon nanomaterial (black cylinders) modified with metallic nanoparticle electrocatalysts (golden spheres) and bound by a layer of anionically charged Nafion polymer (green lines). Also shown is (c) the concept explored here, where a charged Self-Assembled Monolayer (SAM) is used to molecularly increase electrocatalytic ability by the introduction of functional headgroups (purple spheres).

Thermocells with a temperature difference across the two electrodes can convert some heat flux to a flow of electricity through an external circuit.^5^Fig. 1(a) displays a schematic of a thermocell in operation. This is driven primarily due to the entropy difference between two redox states;^4^ diffusion of these species can ensure a constant flow of current, to act as a power generator, or frustrated diffusion can achieve power storage (as a thermocapacitor).^6,7^ Modification to boost entropy differences can boost voltage and therefore electrical power;^8,9^ modification of the electrodes is often performed to boost current and therefore also electrical power.^10,11^ The former entropy differences are typically independent of kinetics, but the latter current is highly sensitive to electron transfer kinetics.^12^

Methods to boost the entropy include coulombic ‘charge additivity’, *i.e.* physically grafting charged moieties adjacent to redox centres.^13,14^ It also includes phase changes^2,10^ and supramolecular association.^2,10^

Modification of the electrodes typically aims to increase surface area and/or enhance the rate of electron transfer. For this reason the application of nanomaterials to thermocell electrodes has been extensively explored,^8,15–17^ often with the further addition of electrocatalytic noble metals such as Pt.^15,18^Fig. 1(b) displays this scenario for a hypothetical carbon nanomaterial decorated with a precious metal nanoparticles, and dropcast with Nafion ionomer to serve as a physical binder to improve the dispersion and stability of the layer.^19^

Some preliminary experiments performed by us observed a significant but temporary boost in thermocell performance for the Fe^2+/3+^ redox couple when electrodes were modified by dropcasting a Nafion/nanomaterial suspension. This was traced back to the anionically-charged Nafion polymer, which was gradually lost from the electrode surface (likely due to desorption and dissolution). While Nafion can undeniably improve the dispersion, stability and charge conduction paths of electrocatalytic materials,^20^ Nafion can also weakly adsorb on electrode surfaces and inhibit electron transfer.^21,22^ It can also act as a non-innocent binder by altering the redox state of bound materials.^23^ Since Nafion was beneficial for Fe^2+/3+^ but had no effect upon [Fe(CN)_6_]^3−/4−^, we speculated this could correspond to enhanced electrostatic interactions between the highly charged redox couples and the electrode surface. In order to investigate this apparent ‘electrostatic electrocatalysis’ phenomena detected in the thermocell, a more rigorous protocol was developed to probe this, based upon self-assembled monolayers.

Self-assembled monolayers (or SAMs) are spontaneously formed molecular assemblies at a surface, typically a metal.^24,25^ This work utilised the well-established affinity of thiol moieties to form SAMs on gold electrodes, *via* Au–S bonds.^24,25^ Some prior investigations have been performed into the influence of the charge of SAM head groups on electrochemical response and rates of electron transfer. For example, ionisable thiol monolayers were investigated and the apparent rate of electron transfer for [Ru(NH_3_)_6_]^3+/2+^ was 300 times faster at R–COO^−^ head groups compared to R–COOH ones, although both were slower than unmodified gold.^26^ Positively charged monolayers significantly hindered [Ru(NH_3_)_6_]^3+^ reduction but only slightly hindered [Fe(CN)_6_]^3−^ reduction; the opposite trend was observed for anionically charged monolayers.^27^ In the above studies, these observations were partially attributed to altered potential drops across the electrical double layer, and primarily due to electrostatically-induced changes in the concentration of redox species near the electrode surface. Electrostatic catalysis of non-redox processes is also known.^28^

Similar results have been observed beyond SAMs, *e.g.* 4-carboxylphenyl groups grafted onto glassy carbon electrodes displayed no voltammetric response for [Fe(CN)_6_]^3−/4−^, but maintained redox features for [Ru(NH_3_)_6_]^3+/2+^ (albeit at slower electron transfer rates than with the bare electrode).^29^ Similar pH-dependent effects have been observed for graphene nanoflakes with carboxylic acid functionalities.^30^

## Results and discussion

### Cyclic voltammetric and impedance spectroscopy analysis of different SAMs

**Fig. 2:**
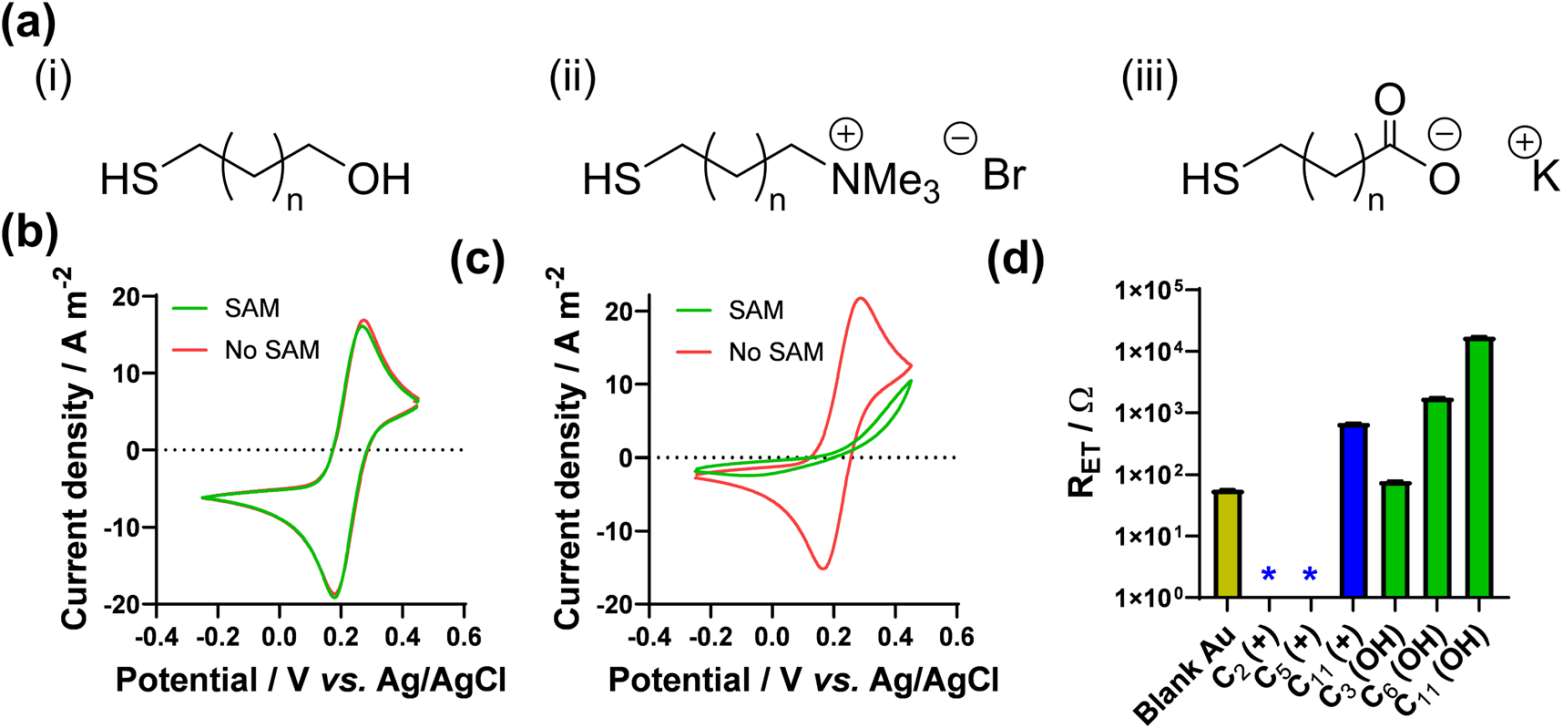
Showing (a) the general chemical structure of the 3 types of thiol-alkyl molecules utilised to form electrostatic SAM layers on Au surfaces, where n is between 0 and 9; the CV responses for (b) 10 mM [Fe(CN)_6_]^3−^ with the 11-carbon anionic SAM and (c) 10 mM [Fe(CN)_6_]^4−^ with the 11-carbon cationic SAM, showing scans with SAM modification (green) and without (red) on the Au electrode; (d) measured apparent electron transfer resistance (*R*_ET_) for 10 mM [Fe(CN)_6_]^3−/4−^, as a function of SAM lengths with cationic and neutral SAMs, where the * indicates an *R*_ET_ couldn't be accurately measured and can be ascribed as ≪10 Ω. Further information on the Nyquist fitting (including frequency ranges) can be found in the ESI.†

As noted above, Nafion ionomer on the surface of electrodes appeared to result in a temporary but significant boost in thermocell performance for the Fe^2+/3+^ redox couple; hence experiments were designed to use self-assembled monolayers (SAMs) to introduce ‘permanent’ coulombic charges close to the electrode surface. SAM layers were achieved through the use of thio-alkyl-head group molecules, which were purchased or synthesised to carry either a cationic ([RNMe_3_]^+^), anionic ([RCOO]^−^) or neutral (OH) head group, to emulate the electrostatic effect temporarily observed for Nafion. The utilised SAMs are summarised in Fig. 2(a) (see ESI,† for a full range of chemical structures in Fig. S1,† as well as synthesis and methods). These SAMs allowed us to evaluate the potential of ‘electrostatic electrocatalysis’ in thermocells, *via* the concept shown in Fig. 1(c). As a result of pH instability and coordination issues between the Fe^2+/3+^ redox couple and the carboxylate SAM, the [Fe(CN)_6_]^3−/4−^ redox couple was selected as a model electrolyte. Finally, the length of the alkyl chain in the SAM was varied with ‘short’ (2 to 3 carbons), ‘medium’ (5 to 7 carbons) and ‘long’ (11 carbons) as additional variables.

The effect of SAMs was initially investigated here using cyclic voltammetry (CV). SAMs were grown for 4 h on Au voltammetry electrodes, and CVs measured separately in both 10 mM K_3_[Fe(CN)_6_] and 10 mM K_4_[Fe(CN)_6_] (both with 1 M KCl supporting electrolyte; full discussion and data in the ESI†). As shown by the CVs (Fig. S2†), significant decreases in the apparent rate of electron transfer was observed for neutral SAMs with chain lengths of 8 carbons or greater, and 6 carbons or greater for the anionic SAM; Fig. 2(b) displays the complete loss of apparent redox chemistry when the 11-carbon anionic SAM was present. This is in agreement with extensive prior literature which has observed slower rates of electron transfer through SAMs for every methylene group,^31–33^*e.g.* [Ru(NH_3_)_6_]^2+/3+^ exhibited slower electron transfer through longer SAMs made from amino-1-alkanethiol, with a complete loss of faradaic peaks by the 11 carbon SAM.^34^ Contrary to the above, in this study the voltammetric response of [Fe(CN)_6_]^3−/4−^ was found to be almost unaffected in the presence of the cationic SAM layers, even up to 11 carbons, as shown in Fig. 2(c). This demonstrates a clear electrostatic effect.

**Fig. 3:**
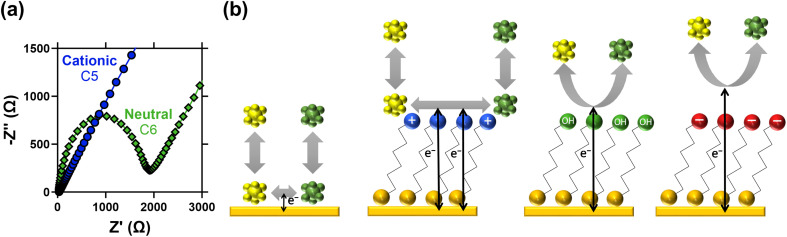
Showing (a) Nyquist plots for the 5-carbon cationic and 6-carbon neutral SAMs (same experimental conditions as Fig. 2(d)), demonstrating the semi-circle electron transfer resistance and linear mass transport resistance components for the neutral SAM, but only mass transport could be observed for the cationic SAMs. Also (b) diagrams representing the likely electron transfer scenarios for the different systems. [Fe(CN)_6_]^3−/4−^ is a known *quasi*-outer sphere electron transfer redox couple,^31^ hence adsorption at the bare Au is potentially a key electron transfer mechanism. This is frustrated at the neutral and anionic SAMs, with the electron forced to quantum tunnel over an extended distance. For the cationic SAM, adsorption of [Fe(CN)_6_]^3−/4−^ at the cationic SAM head groups is likely, resulting in a greater residence time near the electrode surface and thus the significantly decreased electron transfer resistance.

The CV results were followed by more quantitative electrochemical impedance spectroscopy (EIS) analysis, in 10 mM [Fe(CN)_6_]^3−/4−^ (where both ions were present in a 50 : 50 ratio). The solution resistance (*R*_S_) and electron transfer resistance (*R*_ET_) were quantified in the absence of a SAM, and with various lengths of neutral and cationic SAMs. All raw data, fittings and tabulated values are available in the ESI; Fig. 2(d) visually summarises the *R*_ET_ values. The unmodified gold electrode surface was found to have an *R*_ET_ of 56.9 ± 0.8 Ω, and for neutral SAMs with 3-, 6- and 11-carbons the *R*_ET_ increased to 79.2 ± 0.3 Ω then 1776 ± 4 Ω and 17 300 ± 200 Ω, respectively. However, for all cationic SAMs a significant electrocatalytic improvement towards the [Fe(CN)_6_]^3−/4−^ redox couple was observed. The cationic 11-carbon SAM was found to have an *R*_ET_ of 682 ± 5 Ω, which is more than two orders of magnitude lower than the neutral 11-carbon SAM. For the 2- and 5-carbon cationic SAMs the *R*_ET_ component of the Nyquist plots were too small to be fitted, but for both *R*_ET_ ≪ 10 Ω. Example Nyquist plots are shown in Fig. 3(a), clearly showing the electron transfer and mass transfer components were observed when a 6-carbon neutral SAM was present, but electron transfer resistances were too small to be measured (even at higher frequencies) for cationic SAMs shorter than 11 carbons.

The detrimental steric effect of the neutral SAMs, the significant detrimental electrostatic effect of the negative SAMs, and the significant beneficial electrostatic electrocatalytic effect of the cationic SAMs were therefore all confirmed. A schematic of the different scenarios is shown in Fig. 3(b); the neutral SAM necessitates quantum tunnelling of electrons over greater distances, thus lowering the rate of electron transfer.^31–33^ This distance is even greater for the anionic SAMs due to electrostatic repulsion. Beneficial electrostatic electrocatalysis has been previously linked to altered potential differences at the interface, and partitioning to accumulate higher concentrations of ionic species at the interface;^35^ both are possibilities here. Electron transfer can also occur at SAM defect sites^36^ or *via* partition of the redox active species inside the SAM layer^36^ but there is no evidence of this here.

### Effect of different SAMs inside an operational thermocell

Next, this electrocatalysis phenomena was investigated in an operational thermogalvanic cell (or thermocell). Since the thermocell gold electrodes were significantly larger and different from the voltammetry electrodes, some additional optimisation was required to achieve reproducible thermocell results. Primarily, extending the SAM growth time from 4 hours to 24 hours was optimal (Fig. S4†).

For thermocell measurements, two symmetrical gold electrodes were modified with an identical SAM and a temperature difference of 20 K was applied across the cell, as shown by the schematic in Fig. 1(c). The electrolyte used was aqueous 200 mM [Fe(CN)_6_]^3−/4−^, as a 50 : 50 ratio of K_3_[Fe(CN)_6_] and K_4_[Fe(CN)_6_]. This is a non-optimised^12^ and relatively dilute^37^ system, but was employed for proof-of-concept. The steady-state maximum electrical power generated by the cell was measured in line with established techniques.^7^

**Fig. 4:**
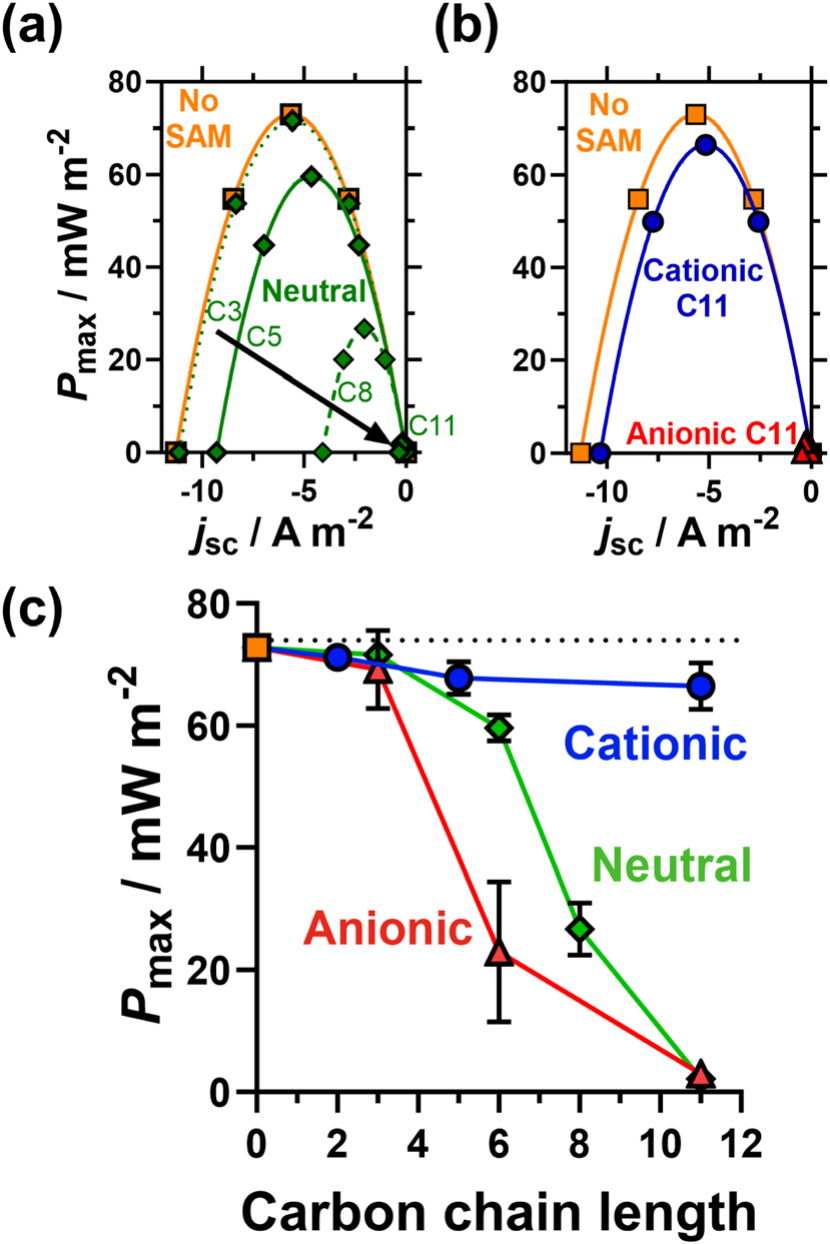
Showing (a) representative power density curves measured for unmodified Au electrodes (■) and electrodes modified with neutral SAMs as a function of chain length (◆), and (b) the 11 carbon cationic (●) and anionic (▲) SAMs. Also (c) the steady-state maximum power, *P*_max_, as a function of SAM head group and chain length. All were measured in thermocells containing 0.2 M K_3_/K_4_[Fe(CN)_6_] (0.1 M of each; applied temperature difference of 20 K). Error bars represent one standard deviation of triplicate measurement; if absent then the error bar was smaller than the data point.

Fig. 4(a and b) plots thermogalvanic power curves as a function of chain length (for the neutral SAMs) and head group charge (for the 11 carbon SAMs); the power generated as a function of carbon chain length and head group is plotted in Fig. 4(c). Interestingly, the voltage generated as a function of temperature difference, or thermogalvanic Seebeck coefficient, remained constant and was entirely unaffected by the presence of SAMs, thus all changes in electrical power were solely due to current differences. The unmodified electrode generated the highest thermogalvanic power, with *P*_max_ = 72.8 ± 1.0 mW m^−2^. Despite the clear electrostatic electrocatalysis evidenced earlier for the shorter cationic SAMs, the power actually decreased slightly, to 71.2 ± 0.9 mW m^−2^ for 2 carbons, 67.8 ± 2.7 mW m^−2^ for 5 carbons and 66.5 ± 3.8 mW m^−2^ for 11 carbons. The major resistance in this thermocell is expected to be mass transport rather than kinetic,^37^ hence this could relate to mass transport phenomena such as slower removal of negatively charged redox products from the electrodes surface. Nevertheless, the power remains largely unchanged whereas for the neutral and anionic SAMs the power drops significantly with increasing chain length. This proves the viability of electrostatic electrocatalysis in a thermocell, which could be highly beneficial to other systems (discussed at the end).

### Evaluating a SAM's ability to improve long-term electrode stability

Finally, gold electrodes in conjunction with [Fe(CN)_6_]^3−/4−^ are well known to suffer from instability issues, including various electron transfer mechanisms, rates and corrosion.^38,39^ This includes in thermogalvanic cells, where initial electrocatalysis by gold nanoparticles was previously observed to result in poor performance after moderate operation times (relative to just graphite) due to passivating layers of Au/[Fe(CN)_6_]^3−/4−^ reaction products forming.^40^ However, in this study no evidence of this was observed when SAMs were present, so the ability of cationic SAMs to increase the longevity and stability of the thermocell system was explored. SAMs are in fact known to afford the underlying metal electrodes some protection against corrosion, with cyanide known as a useful probe molecule to test the quality of SAM formation;^41^ Electrochemical Scanning Tunnelling Microscopy of Au(111) protected with hexadecyl mercaptan SAMs have shown significantly reduced CN^−^-induced corrosion;^42^ this also applied to aqueous Br^−^ solutions, with a range of techniques demonstrating OH- and COOH-terminated SAMs provided even more corrosion protection than CH_3_-terminated SAMs, but also changing the corrosion mechanism (pitting *vs.* layer-by-layer, respectively).^43^

Since this study has not investigated the Au electrode surface in detail, we can only discuss here the ability of the SAMs to achieve protection against ‘kinetic passivation’, or observed decreases in the electrocatalytic ability of the Au electrodes by extended exposure to concentrated [Fe(CN)_6_]^3−/4−^. This was quantitatively demonstrated both electrochemically and thermoelectrochemically, by using EIS to measure *R*_ET_, and monitoring the thermogalvanic power density. Both voltammetry Au electrodes and thermocell Au electrodes were cleaned, measured, soaked in a [Fe(CN)_6_]^3−/4−^ solution for 24 hours, and then re-measured. Here the thermocell electrolyte was changed from 200 mM [Fe(CN)_6_]^3−/4−^ to 400 mM to increase it's ability to corrode the gold, and this was also used as the soaking solution.

**Fig. 5:**
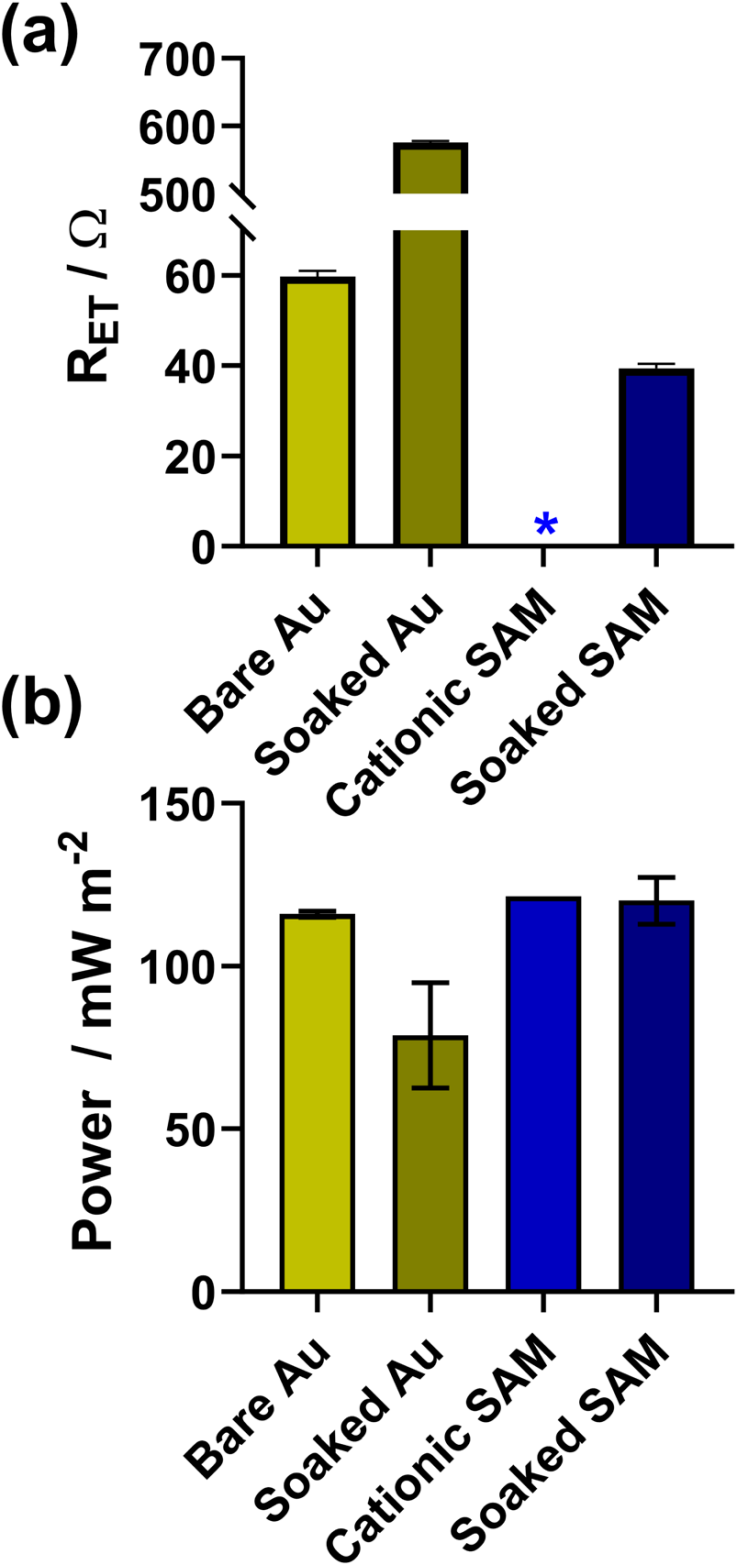
Bar charts showing measurements for Au electrodes with and without the 2-carbon cationic SAM, before and after soaking in 0.4 M K_3_/K_4_[Fe(CN)_6_] for 24 h. Panel (a) shows the apparent electron transfer resistance (*R*_ET_) measured for the voltammetric Au electrode in 10 mM K_3_/K_4_[Fe(CN)_6_] with 1 M KCl supporting electrolyte; (b) shows the thermogalvanic *P*_max_ measured in the thermocell with 0.4 M K_3_/K_4_[Fe(CN)_6_]. The * indicates the *R*_ET_ was too small to be measured. Further information relating to the Nyquist fitting frequency ranges can be found in Fig. S10.†

As shown in Fig. 5(a) and (b), the bare electrodes were notably poisoned, with the *R*_ET_ increasing by an order of magnitude and the power dropping by *ca.* 32% (from 116 ± 1 mW m^−2^ to 79 ± 16 mW m^−2^). Conversely, for electrodes modified with 2 carbon cationic SAMs, the *R*_ET_ before soaking was too small to be measured, and afterwards this increased to 39.4 ± 1.0 Ω (the unmodified electrode had an *R*_ET_ of 59.8 ± 1.2 Ω), although it's unclear if this is due to partial kinetic passivation of the Au, or if the SAM underwent partial restructuring. However, the thermogalvanic power for the SAM-modified electrodes (121 ± 1 mW m^−2^) in this more concentrated electrolyte not only exceed the power of the bare electrodes (116 ± 1 mW m^−2^), but also remained largely unchanged despite 24 h soaking in [Fe(CN)_6_]^3−/4−^ (120 ± 7 mW m^−2^), clearly indicating enhanced electrocatalysis and no significant kinetic passivation of the thermocell electrode surface.

### Further potential applications of this ‘electrostatic electrocatalysis’ and enhanced electrode stability

Ultimately this study elegantly demonstrates the concept of how molecular electrostatic electrocatalysis can be applied to thermogalvanic cells. This is significant because, whereas previous attempts to use gold nanomaterials to enhance electrocatalysis has also decreased stability (due to the increased reactivity of the nanostructured gold), this study demonstrates how appropriately electrostatically charged SAMs can lower the apparent electron transfer resistance and simultaneously improve long-term stability. This is critical when considering most industrial waste heat applications have commercial payback periods of 3 to 15 years,^44^ demonstrating the time frames thermocell devices should continuously operate for (and ideally exceed).

Thermogalvanic cells routinely employ highly ionically charged redox couples and operate at relatively small overpotentials, hence are especially positioned to benefit from electrostatic electrocatalysis. While this concept has been demonstrated here, the particular experimental setup of gold and [Fe(CN)_6_]^3−/4−^ isn't able to significantly benefit from it. Notably, gold is already an excellent electrocatalyst and in this case is even superior to platinum (Fig. S11†), [Fe(CN)_6_]^3−/4−^ is relatively kinetically fast, and the bulk thermocell used for measurements is primarily mass transport-limited rather than kinetically-limited. However, the ultimate goal is for this demonstrated concept to be extended to improving cheaper electrodes with more sustainable redox couples (both of which typically introduce kinetic issues^3,4^). For example inexpensive (nano)carbon electrodes could be electrostatically modified with *e.g.* covalently grafted charged groups,^45^ in order to improve their electrocatalytic ability towards more sustainable redox couples *e.g.* kinetically slower but highly charged Fe^2+/3+^ salts.^46,47^ The results from this study indicate such modification is expected to significantly improve both thermocell power output (more so than could be observed here using Au as the base material) and potentially the effective lifetime.

## Conclusions

In summary, our study reveals that electrostatic electrocatalysis can play a crucial role in the performance of thermogalvanic cells. Spurred on by negatively-charged Nafion's ability to temporarily enhance Fe^2+/3+^ cell performance, but hindered by its instability and irreproducibility, we employed gold electrodes with various self-assembled monolayers and explored their impact on electron transfer and power generation. Using [Fe(CN)_6_]^3−/4−^ as a model redox couple, cationic SAMs showed significant electrocatalytic improvement, while neutral and anionic SAMs exhibited detrimental kinetic effects. SAMs also afforded the electrodes long-term protection against established corrosion and passivation issues.^6^ These findings demonstrate the general potential of electrostatic electrocatalysis, and more specifically how this concept could be applied to enhance the performance of sustainable, cost-effective electrodes in thermogalvanic cells, paving the way for long-term, efficient operation. This could also be extended to other systems with highly concentrated, charged electrolytes that require long-term effective electrocatalysis, *e.g.* carbon felt electrodes in redox flow batteries.

## Experimental

Below is a brief experimental section containing the key pertinent information. A much more comprehensive and detailed Experimental section can be found in the ESI.†

### Cyclic voltammetry (CV) and electrochemical impedance spectroscopy (EIS)

All CV and EIS experiments were carried out using a PGSTAT204 potentiostat with NOVA software (Metrohm, UK). The electrochemical setup was a 1.6 mm diameter Au disc working electrode, a Pt counter electrode, and an Ag/AgCl (3 M NaCl) reference electrode (all BASi, USA). All scans were, unless specified otherwise, recorded *ex situ* to the thermocell and at ambient temperature (*ca.* 22 °C). CVs had a scan rate of 100 mV s^−1^; impedance spectra were initially recorded from 20 000 to 0.1 Hz with an amplitude of 10 mV, and the frequency range adjusted if required.

### Thermoelectrochemistry

All thermoelectrochemical measurements were performed using a two-chamber thermocell, which is detailed in the ESI† and has been reported elsewhere.^7,12^ The electrodes were solid gold discs (99.99% pure, 1 mm thick discs with 10 mm diameter, from Surepure Chemetals, USA) with an electrode surface area of 35 mm^2^ exposed to the electrolyte and an inter-electrode spacing of 7.4 mm. The outside of the electrodes had an applied temperature difference, Δ*T*, of 20 K (hot side 45 °C, cold side 25 °C) which has previously been demonstrated^7,12^ to result in an ‘experienced’ temperature difference of *ca.* 18 K, due to the thickness of the electrodes. All potential, current and power measurements were performed using a Keysight B2901A Source Measure Unit and Quick IV software (Keysight, UK), and were carefully measured and allowed to reach steady-state, following precisely the ‘sequence of constant voltages’ method previously reported.^7,12^

### SAM growth

All SAM forming molecules were (i) thiol, (ii) thioacetate or (iii) isothiouronium-based (see ESI† for full details).

For growth on the 1.6 mm diameter Au disk electrode for CV and EIS measurements, the optimised protocol was found to be a solvent composition of ethanol : dichloromethane (EtOH : DCM, 9 : 1), with incubation 4 hours before being rinsed with EtOH and dried under a stream of N_2_. A volume of 1 mL was used, with a concentration of (i) 1 mM thiol, (ii) 1 mM thioacetate or (iii) 1 mM isothiouronium and 0.9 mM KOH; the *in situ* hydrolysis of the isothiouronium to the free thiol was found to be optimal, compared to attempting to isolate the free thiol and then utilise.

For thermoelectrochemistry, the SAMs were grown on two significantly larger 10 mm diameter Au electrodes. Given the *ca.* order of magnitude higher surface area, the volume was initially increased to 10 mL EtOH : DCM (9 : 1); however, in order to get reproducible SAM formation it was also necessary to increase the concentration to 5 mM thiol, 5 mM thioacetate or 5 mM isothiouronium (the latter with 4.5 mM KOH), before being rinsed with EtOH and dried under a stream of N_2_. The incubation duration was also varied between 3 and 24 hours (results shown in the ESI†) with 24 hours found to be optimal.

## Data availability

The datasets supporting this article have been uploaded as part of the ESI† (*e.g.* graphically reported values are all tabulated).

## Author contributions

All: writing; visualisation. KL: methodology; investigation. MB: validation; investigation. LA: conceptualisation; supervision; funding acquisition.

## Conflicts of interest

There are no conflicts to declare.

## Supplementary Material

SC-015-D3SC06766A-s001
