# Supplementary material for: Self-assembled monolayers for electrostatic electrocatalysis and enhanced electrode stability in thermogalvanic cells
Source: Chem Sci. 2024 Apr 3;15(18):6958–64. doi: 10.1039/d3sc06766a (PMC11077577; doi:10.1039/d3sc06766a)
Supplement: SC-015-D3SC06766A-s001 [file SC-015-D3SC06766A-s001.pdf]

Electronic Supplementary Material (ESI) for Chemical Science.
This journal is © The Royal Society of Chemistry 2024

Electronic Supporting Information for:

Self-Assembled Monolayers for Electrostatic Electrocatalysis and Enhanced Electrode Stability in Thermogalvanic Cells

Kristine Laws[a], Mark A. Buckingham,[a,b] and Leigh Aldous[a,*]

[a] Department of Chemistry, Britannia House, King's College London, London, SE1 1DB, UK

[b] Present Address: Department of Materials, The University of Manchester, Oxford Road, Manchester M13 9PL, UK.

* Corresponding author: leigh.aldous@kcl.ac.uk

Contents

Experimental 3

Chemicals 3

Synthesis of 2-(5-trimethylammoniumpentyl)isothiouronium dibromide 3

Synthesis of 2-(10-carboxydecyl)isothiouronium bromide 4

Synthesis of S-(11-trimethylammoniumundecyl)thioacetate 4

SAM Growth 4

Thermoelectrochemistry 5

Cyclic Voltammetry (CV) 6

Electrochemical Impedance Spectroscopy (EIS) 6

SAM-inducing molecules used 8

SAM-layer deposition 9

Impedance analysis of SAM-layers 11

Incubation time study of cationic SAMs 13

Power density of SAM layers 14

Comparative Nyquist plots 15

Impedance fitting model and fitted Nyquist plots 15

SAM protection of Au against passivation – tabulated data of fitted Nyquist plots 19

SAM protection of Au against passivation - Nyquist plots 19

SAM protection of Au against passivation – fitted Nyquist plots 20

Electrocatalytic comparison of unmodified electrode materials (platinum, gold and glassy carbon) 22

Experimental

Chemicals

All chemicals were purchased from UK suppliers and used as received unless otherwise specified. These were: Potassium Ferricyanide (III) (MP Biomedicals, 99%), Potassium Ferricyanide (III) (Merck Life Science UK Ltd, 99%), Potassium Hexacyanoferate (II) Trihydrate (Merck Life Science UK Ltd, 99%), 3-Mercaptopropanoic Acid (Sigma), 3-Mercapto-1-propanol (Merck Life Science UK Ltd, 95%), Acetylthiocholine iodide (Sigma, 98 %), (5-Bromopentyl)trimethylammonium bromide (Merck Life Science UK Ltd), 6-Mercaptohexanoic acid (Merck Life Science UK Ltd, 90%), Mercapto-1-hexanol (Merck Life Science UK Ltd, 97%), 8-Mercapto-1-octanol (Merck Life Science UK Ltd), 11-Bromoundecanoic Acid (Sigma, 99 %), 11-Mercapto-1-undecanol (Merck Life Science UK Ltd, 97%), S-(11-Bromoundecyl)thioacetate (Santa Cruz biotechnology), Thiourea (Sigma, >99 %), Potassium hydroxide (pellets, VWR).

Synthesis of 2-(5-trimethylammoniumpentyl)isothiouronium dibromide

(5-Bromopentyl)trimethylammonium bromide (1 g, 3.46 mmol, 1 eq.) was added in one portion to a solution of thiourea (263 mg, 3.77 mmol, 1 eq.) in dry EtOH (10 mL). The reaction was left stirring at room temperature for 24 h. The solvent was removed in vacuum and the solid residue and dried under vacuum. Quantitative conversion was confirmed by ^{1}H-NMR.

Synthesis of 2-(10-carboxydecyl)isothiouronium bromide

11-Bromoundecanoic Acid (1 g, 3.77 mmol, 1 equivalence) was added in one portion to a solution of thiourea (287 mg, 3.77 mmol, 1 equivalence) in dry EtOH (10 mL). The reaction

was left stirring at room temperature for 24 h. The solvent was removed in vacuum and the solid residue and dried under vacuum. Quantitative conversion was confirmed by ^{1}H-NMR.

Synthesis of S-(11-trimethylammoniumundecyl)thioacetate

Trimethylamine (4.2 M in EtOH, 0.4 mL, 1.62 mmol, 1 equivalence) was added dropwise to S-(11-Bromoundecyl)thioacetate (0.5 mL, 0.5 g, 1.62 mmol, 1 equivalence) was dissolved in dry EtOH (10 mL). The reaction was left stirring at room temperature for 24 h. The solvent was removed in vacuum and the solid residue and dried under vacuum. Quantitative conversion was confirmed by ^{1}H-NMR.

SAM Growth

The optimised protocol for SAM growth was found to be a solvent composition of ethanol:dichloromethane (EtOH:DCM, 9:1), with incubation 4 hours before being rinsed with copious amounts of EtOH and dried under a stream of N_2. All preparation and incubation were carried out in the absence of light, and the solutions were kept in the dark.

The solutions used a concentration of either (i) 1 mM thiol, (ii) 1 mM thioacetate or (iii) 1 mM isothiouronium and 0.9 mM KOH; the *in situ* hydrolysis of the isothiouronium to the free thiol was found to be optimal, compared to attempting to initially purify and isolate the free thiol, and then use for SAM growth.

The SAM layers were grown on 2 different gold electrodes depending on their use, and since the electrodes had very different geometries different assemblies were used (see Figure S1a).

For cyclic voltammetry and impedance spectroscopy, the SAMs were grown on standard Au electrodes for Voltammetry (1.6 mm diameter, BASi, USA). These electrodes are 75 mm long, 6.4 mm wide cylinders of PEEK, with a 1.6 mm diameter Au disk at one end. The electrode was suspended in 1 mL of 1 mM solution to allow SAM growth, protected from light. Using a 15 mL glass sample vial with an appropriately sized hole drilled in the lid allowed the system to be sealed, and have the Au electrode tip suspended at half-depth submerged in the solution.

For thermoelectrochemistry measurements, the SAMs were grown on significantly larger Au electrodes (disc electrodes, 99.99% pure, 1 mm thick with 10 mm diameter, from Surepure Chemetals, USA). Since these gold disks were entirely gold and the measurement required two electrodes, this was achieved by incubating two electrodes together in a sealed glass Petri dish to allow the SAM to grow on the electrode. All polishing and handling of the Au disks was achieved using round tip lock hold tweezers, to handle the discs around their circumference. Given the *ca.* order of magnitude higher surface area, the solution volume was initially increased from 1 mL to 10 mL EtOH:DCM (9:1) while concentration was maintained at 1 mM. However, only small and variable differences were observed between SAM and no-SAM electrode measurements. In order to get reproducible SAM formation it was found necessary to use 10 mL and also increase the concentration to 5 mM thiol, 5 mM thioacetate or 5 mM isothiouronium (the latter with 4.5 mM KOH). The incubation duration was also varied between 3 and 24 hours (results presented below). Once removed from the incubation solution, the electrodes were rinsed with copious amounts of EtOH and dried under a stream of N_2. The side of the disk facing downwards into the glass Petri dish was assumed to have a poorer quality SAM, so was first polished against Kimwipe paper tissue soaked in ethanol, dried, and in the thermocell this side faced the heat exchangers and was

used to make the electrical connection. The side facing up into the SAM solution was carefully protected from bright light and contact, and in the assembled thermocell it faced the electrolyte.

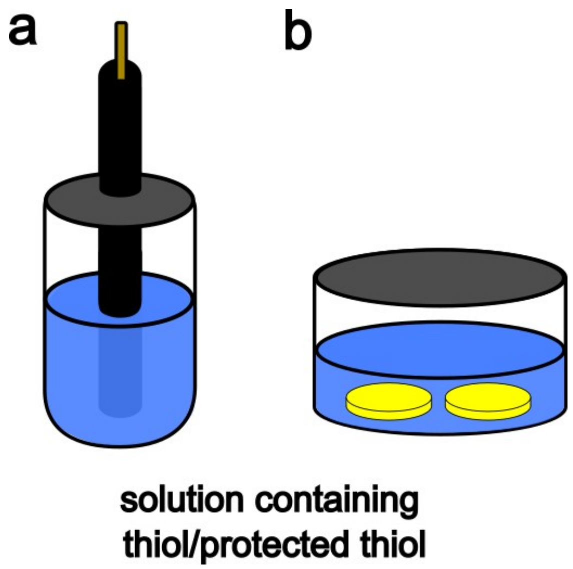

Figure S1: Diagram showing the SAM growth setup utilised for both (a) the BASi Voltammetry PEEK-housed gold disc electrode, and (b) the two solid-gold gold disk electrodes used to assemble the thermocell.

Thermoelectrochemistry

All thermoelectrochemical measurements were performed using a two-chamber tailormade poly(methyl methacrylate) (PMMA) thermocell, which was made in-house. The two-chamber thermocell was machined from a single block of PMMA (30 mm (width) × 20 mm (height) × 8.4 mm (depth)) and has been previously reported in detail elsewhere.[2] Each chamber was a 6.7 mm diameter cylinder (giving a geometric electrode surface area of 35 mm^2) and giving an inter-electrode spacing of 7.4 mm. The electrodes were 10 mm diameter circles which were inserted into 0.5 mm deep lips machined around the chambers in the thermocell, and were solid gold electrodes (gold disc electrodes, 99.99% pure, 1 mm thick discs with 10 mm diameter, from Surepure Chemetals, USA) Temperature control was achieved using copper heat exchangers connected to RS-TX150 thermostatic circulator baths (Grant Instruments

Ltd, UK), as previously described.[2] Notably, a temperature gradient forms between the isothermal water sources and the surfaces of the thermogalvanic electrode, especially given the 1 mm thickness of these gold electrodes; this has been previously characterised for our cell,[3] such that an applied temperature difference, ΔT, of 20 K, equates to an 'experienced' temperature difference of *ca.* 18 K. The applied (rather than experienced) temperature difference is utilised throughout this manuscript. All potential, current and power measurements were performed using a Keysight B2901A Source Measure Unit and Quick IV software (Keysight, UK), were carefully measured and allowed to reach steady-state, following precisely the 'sequence of constant voltages' method previously reported.[3]

Cyclic Voltammetry (CV)

Cyclic voltammetric experiments were carried out using a PGSTAT204 potentiostat with NOVA software (Metrohm, UK). The electrochemical setup was either a 1.6 mm diameter Au disc working electrode when specified, a 1.6 mm diameter Pt disc counter electrode, and an Ag/AgCl (3 M NaCl) reference electrode (all BASi, USA). All scans were recorded at a scan rate of 100 mV s^{-1}, unless specified otherwise. The cyclic voltammetry was performed *ex-situ* to the thermocell and at ambient temperature (*ca.* 22 °C).

Electrochemical Impedance Spectroscopy (EIS)

Electrochemical impedance spectroscopy measurements were carried out using a PGSTAT204 potentiostat with NOVA software (Metrohm, UK). These were performed *ex-situ* to the thermocell at ambient temperature (*ca.* 22 °C) using a 1.6 mm Au disc working electrode, a 1.6 mm diameter Pt disc counter electrode, and an Ag/AgCl (3 M NaCl) reference electrode (all BASi, USA). Impedance spectra were initially recorded from 20,000

to 0.1 Hz with an amplitude of 10 mV; after this initial measurement the range was adjusted if required, which was generally only required for the longest SAM chains.

SAM-inducing molecules used

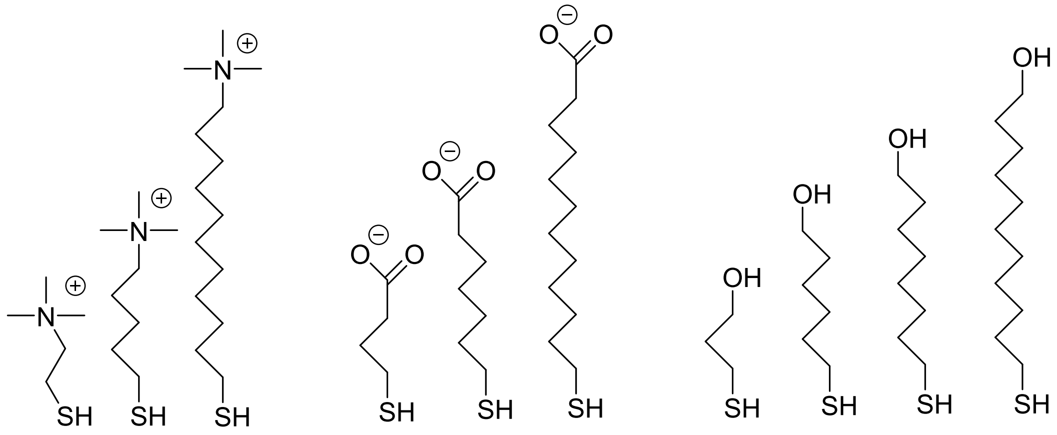

Figure S2. Figure showing relative lengths of investigated SAMs in this study.

SAM-layer deposition

The effect of electrostatic attraction and repulsion was focussed upon by choosing SAM-layer forming molecules that end in either a cationic (ammonium), neutral (using a hydroxyl) or anionic (using a carboxylate) head group. Selection focussed on comparing the effect of both charge on the head group and the alkyl chain lengths. Trimethylammonium was selected as the positive headgroup and deprotonated carboxylic acid as a negative headgroup. Figure S2 shows the cyclic voltammetry of $K_3/K_4[Fe(CN)_6]$ in the presence and absence of different SAMs. The CVs in the presence of the positively charged head group can be seen in Figure S2(a), (b) and (c), with carbon chain lengths of 2, 5 and 11 respectively. Figure S2(d), (e) and (f) show the negatively charged SAMs with carbon chains of 3, 6 and 11, respectively. It can clearly be seen that negative SAMs with chain lengths above 6 are able to reduce and prevent the flow of any current, Figure S2(e) and (f). However, positively charged SAMs (Fig S2(a) – (c)) seem to have no detrimental effect on the redox process, independent of chain length, even up to 11 carbon alkyl chain length, which is also true for the negative C3 SAM. The hydroxyl equivalent (Figure S2(h-j)) also show no significant detrimental effect on the redox process for the 3 and 6 carbon alkyl chain lengths, but above 8 carbon alkyl chain lengths was found to significantly decrease the electrocatalytic ability of the electrode towards the $[Fe(CN)_6]^{3-/4-}$ redox couple.

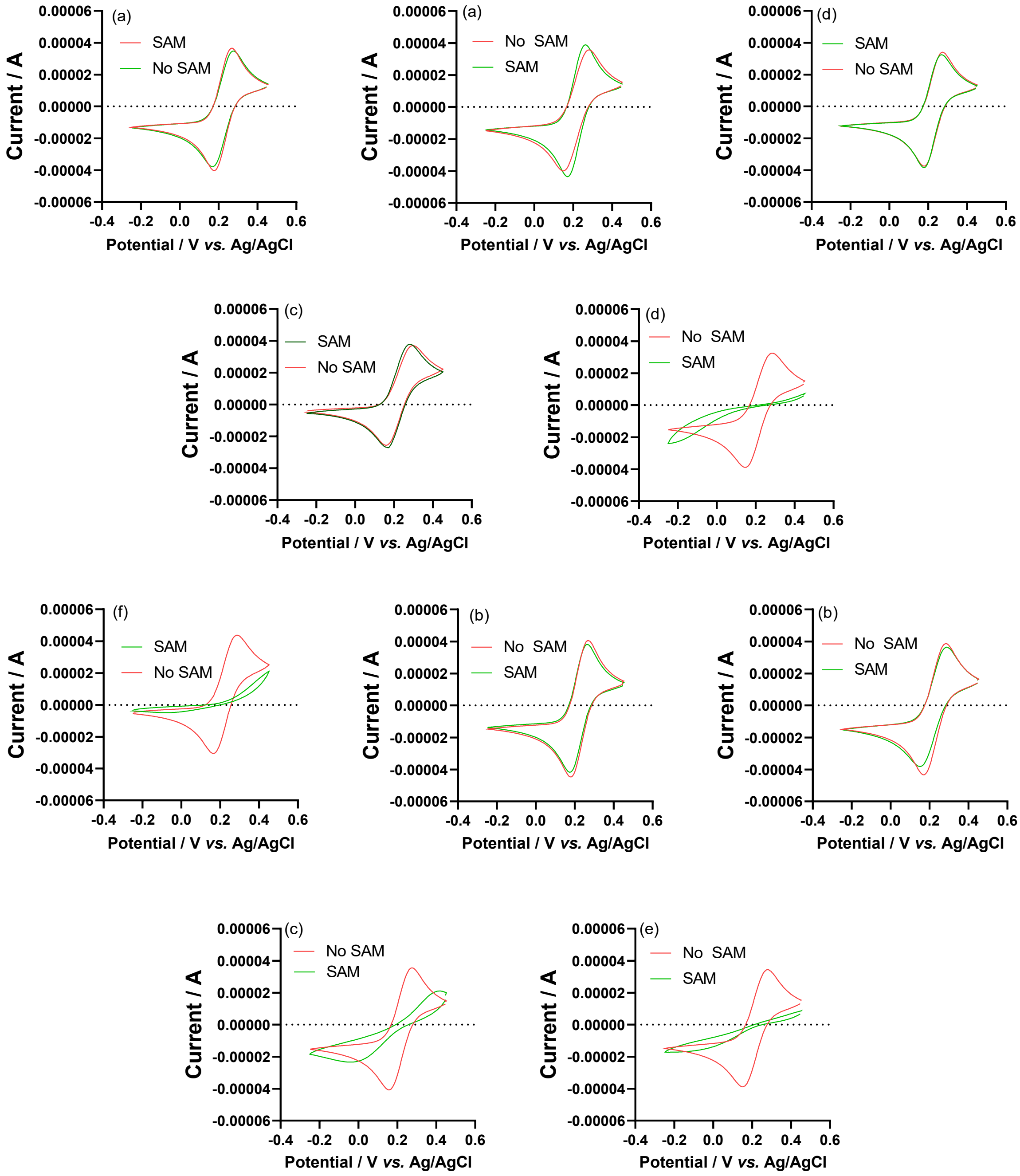

Figure S3 - cyclic voltammetry of $K_3/K_4[Fe(CN)_6]$ (10 mM) on gold in the presence (red) and absence (green) of SAMs after 4 hour incubation. (a) C2 NMe_3^+ (b) C5 NMe_3^+ (c) C11 NMe_3^+ (d) C3 COO^- (e) C6 COO^- (f) C11 COO^- (b) C3 OH (c) C6 OH (d) C8 OH (e) C11 OH

Impedance analysis of SAM-layers

The solution (R_S) and electron transfer (R_{ET}) resistances were measured for a 10 mM $K_3/K_4[Fe(CN)_6]$ solution with 1 M KCl as supporting electrolyte. Figure S3 shows that all systems where the SAM layer was present on the electrode surface were equivalent and had roughly 50% of the solution resistance of the system in the absence of the SAM layer. The reason for this significant benefit with respect to mass transport is not known but could be a consequence of having functional groups present in the localised environment in which EIS is measured.

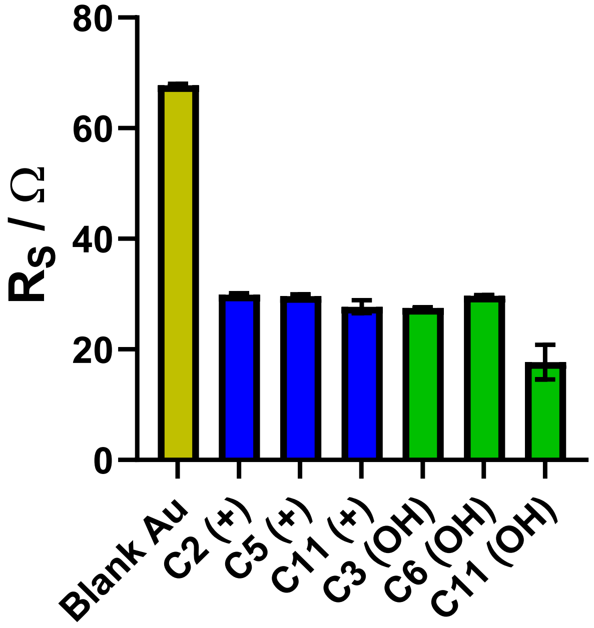

Figure S4. Solution resistance (R_S) values for investigated SAM layers on a gold electrode, compared to the bare electrode surface, using CX (Y) where the X value represents alkyl chain length and the (Y) represents the head group on the SAM.

Table S1 – Table of data for Figure 1(e) and Figure S3.

Alkyl chain length (end group)	Solution resistance (R_S) / Ω	Electron transfer resistance (R_{ET}) / Ω
Blank Au	67.8 ± 0.2	56.9 ± 0.8
C2 (+)	29.9* ± 0.3	<10
C5 (+)	29.6* ± 0.3	<10
C11 (+)	27.7 ± 1.2	682 ± 5
C3 (OH)	27.5 ± 0.1	79.2 ± 0.3
C6 (OH)	29.7 ± 0.2	1,776 ± 4
C11 (OH)	17.7 ± 3.2	17,300 ± 200
* These were for a fixed R_{ET} of 10 Ω; the actual R_{ET} was less than this (discussed later)		

Incubation time study of cationic SAMs

The SAMs were grown on 2 gold electrodes for use in the thermogalvanic cell. Figure S4 shows a graph comparing effect of incubation time for the cationic SAM (C3 triangle, C11 diamond) with the power density. From the CV (Figure S2) it is expected that the cationic SAM would have very little effect on the $K_3/K_4[Fe(CN)_6]$ (0.1 M of each) system and therefore taken to be representative of all the SAM systems. Although the power density changes minimally with time when incubating the C3 SAM, the longer chains were found to take longer to assemble. The C11 SAM appeared to form most effectively after 24 hours; some preliminary experiments (without repeats) were performed at 48 hours, and no significant difference was observed from the 24 h results. From this data it was decided that all the SAMs be incubated for 24 hours to produce the most representative monolayer at all chain lengths.

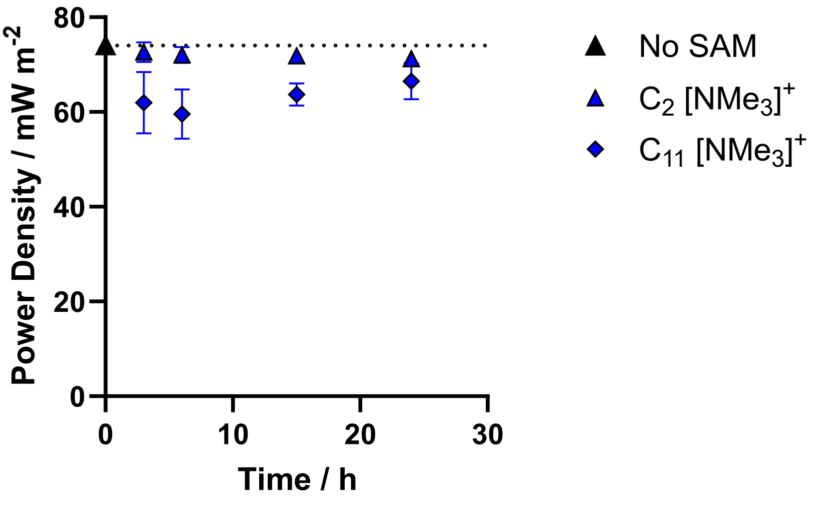

Figure S5 - Graph showing how the power density of a 1:1 $K_3/K_4[Fe(CN)_6]$ thermocell (0.2 M total concentration) in the presence of either C2 cationic SAM (blue circles), C11 cationic SAM (blue diamonds), or the absence of a SAM (black triangle) as a function of the incubation time for SAM growth between 3 h – 24 h.

Power density of SAM layers

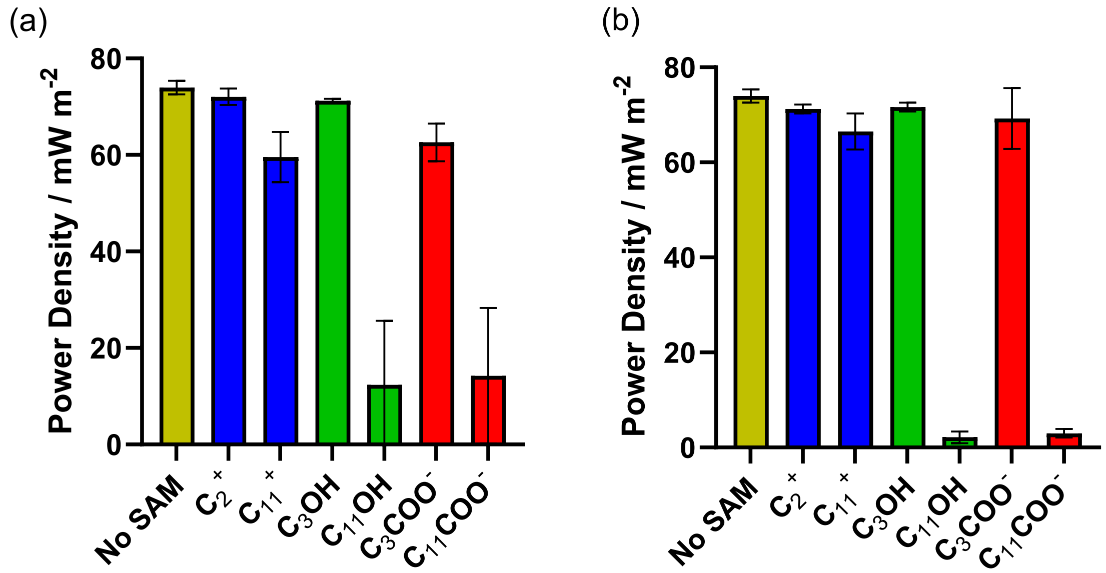

Figure S6 – Bar graphs showing how the power density of a 1:1 $K_3/K_4[Fe(CN)_6]$ thermocell (0.2 M total concentration) in the presence of cationic (NMe_3^+, blue), neutral (OH, green) and anionic (COO^-, red) SAMs, or the absence of a SAM (yellow). These were measured after SAM growth incubation times of (a) 6 h and (b) 24 h.

Comparative Nyquist plots

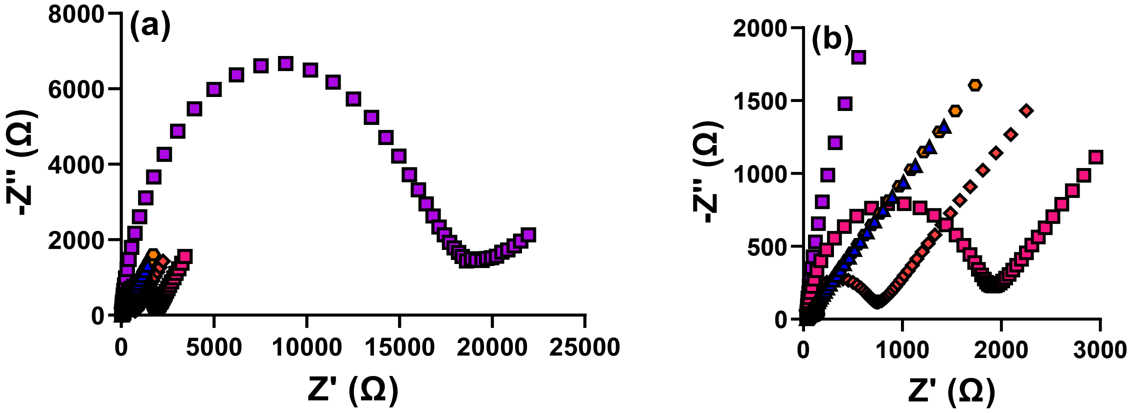

Figure S7. Nyquist plots of all impedance analysis where blank (blue circle), C2 (blue triangle), C5 (orange hexagon) and C11 (red diamond) ($[NMe_3]^+$ end group) and C3 (purple inverted triangle), C6 (pink square) and C11 (purple square) ([OH] end group) are all shown. Information on frequency range used to fit the data is given in Figure S8.

Impedance fitting model and fitted Nyquist plots

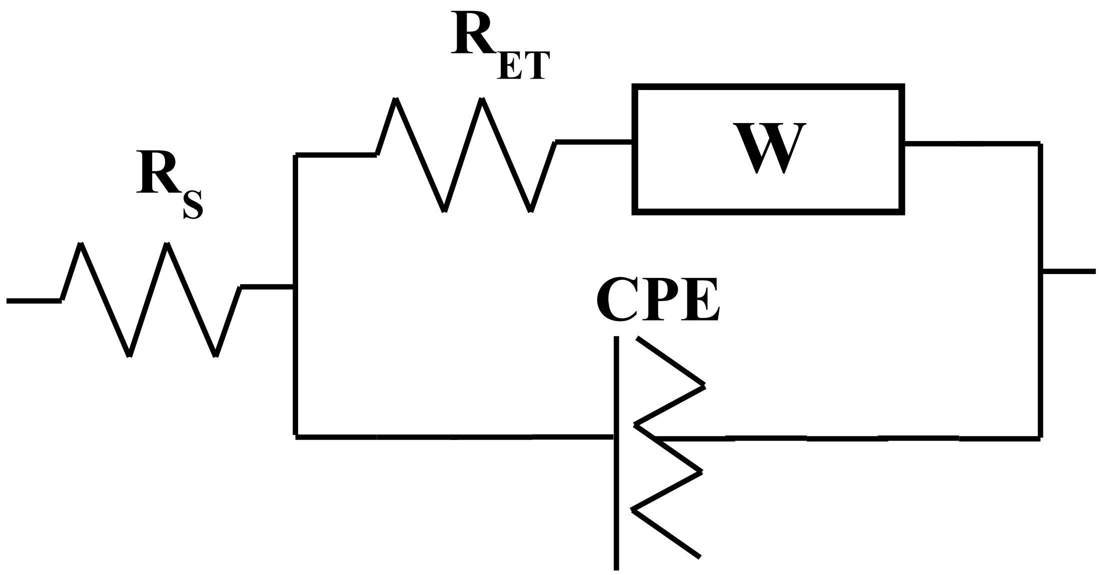

Figure S8. Model used to fit the impedance Nyquist plots in Figures 3 and 4.

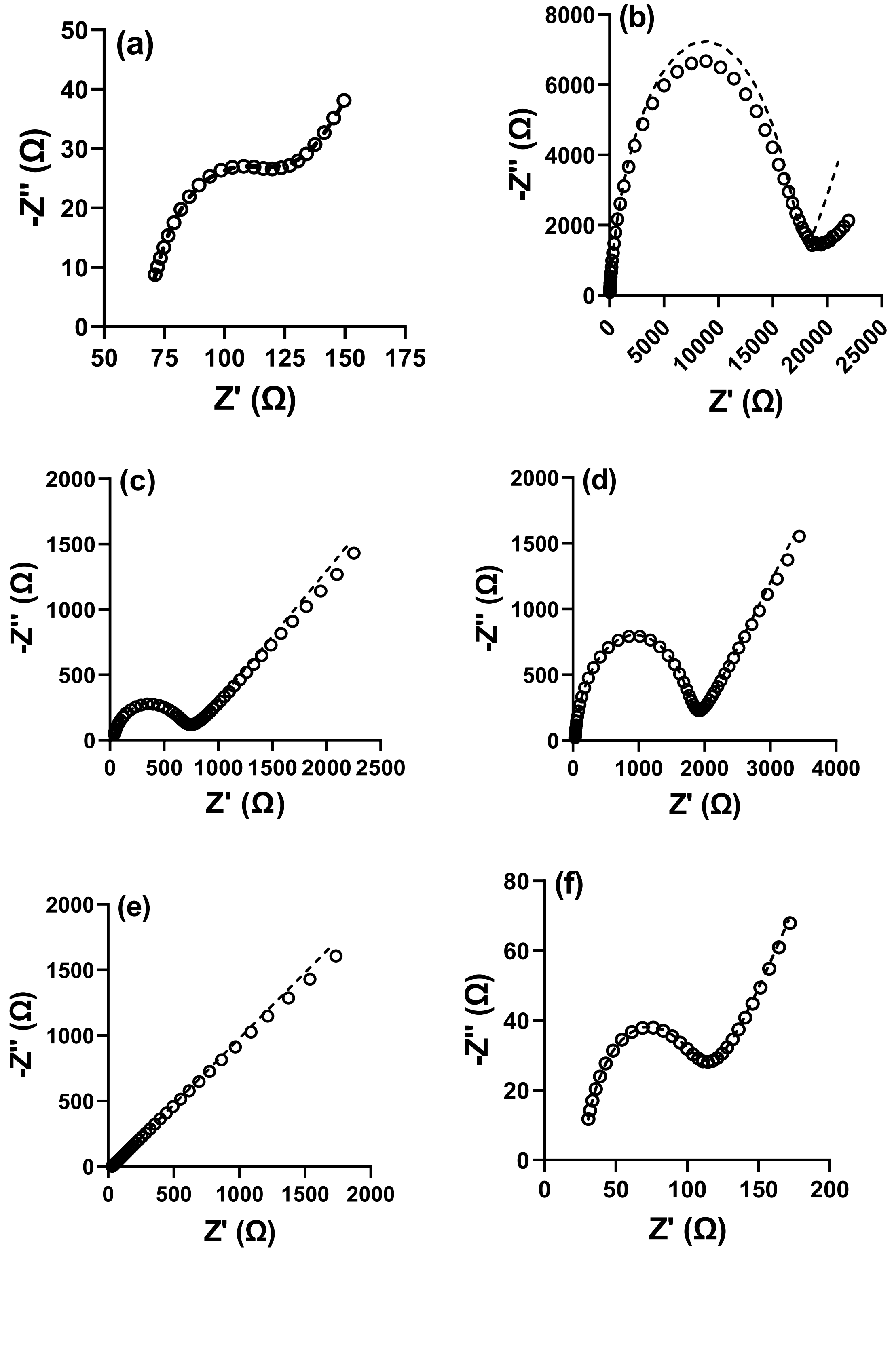

(a)
-Z'' (Ω)
Z' (Ω)
(b)
-Z'' (Ω)
Z' (Ω)
(c)
-Z'' (Ω)
Z' (Ω)
(d)
-Z'' (Ω)
Z' (Ω)
(e)
-Z'' (Ω)
Z' (Ω)
(f)
-Z'' (Ω)
Z' (Ω)

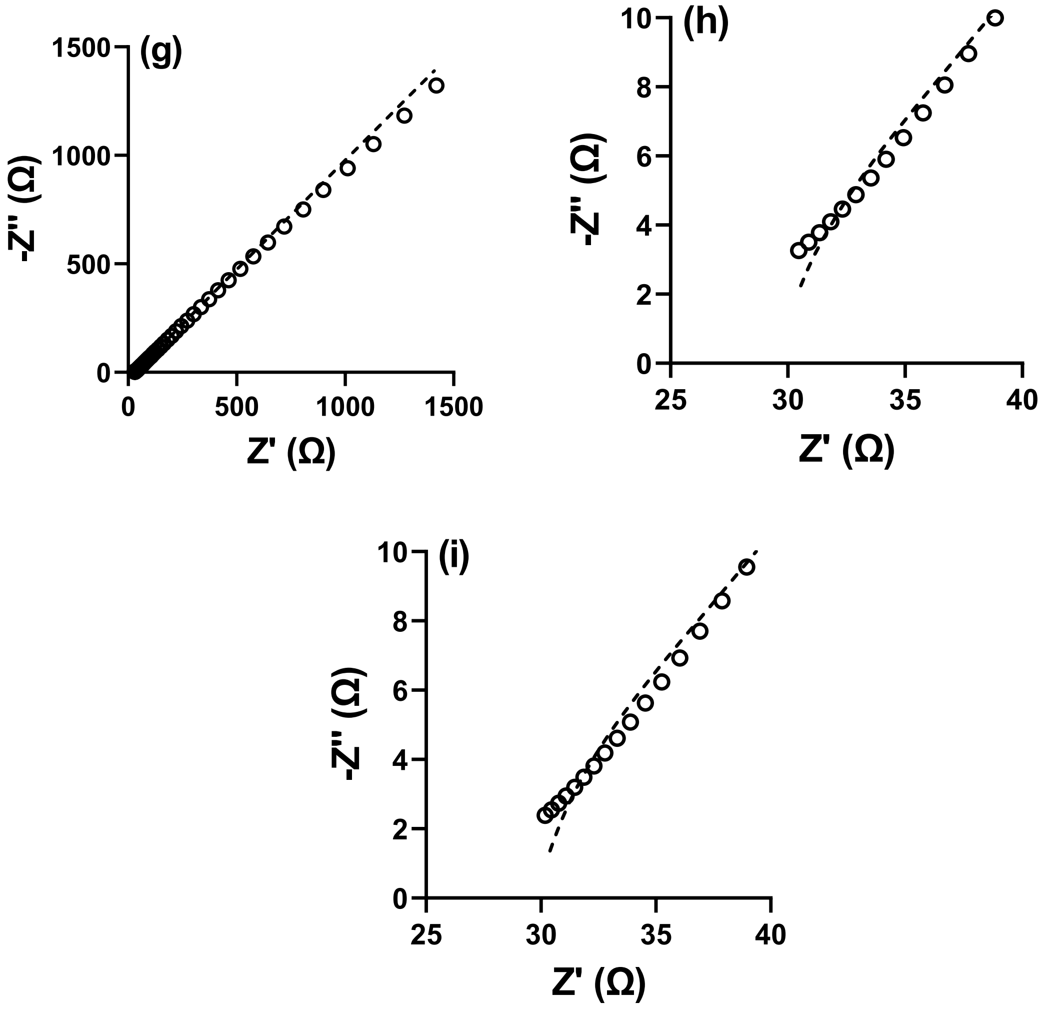

Figure S9 – Experimental (hollow circle) and modelled fitting (dashed line) of the investigated SAMs with various chain length with both cationic $[NMe_3]^+$ and neutral [OH] end groups. Where (a) blank (fit in the range 50 kHz – 224 Hz), (b) C11 (OH, fit in the range 25 kHz – 0.1 Hz), (c) C11 (+, fit in the range 25 kHz – 0.1 Hz), (d) C6 (OH, fit in the range 50 kHz – 0.1 Hz), (e) C5 (+, fit in the range 50 kHz – 0.1 Hz), (f) C3 (OH, fit in the range 50 kHz – 44 Hz) and (g) C2 (+, fit in the range 75 kHz – 0.1 Hz) are all shown. (h) and (i) are 'zoomed in for both (h) C2 and (i) C5 $[NMe_3]^+$ SAMs, with a fixed R_{ET} of 10 Ω.

Table S2: Table showing the thermogalvanic power density (mW m^{-2-}) measured for 1:1 $K_3/K_4[Fe(CN)_6]$ (0.2 M total concentration) with the cationic (NMe_3^+) SAM attached at both electrodes, at various incubation times between 0 h (no SAM) and 24 h.

Time / h<br>Chain Length	0	3	6	15	24
2	73.96 ± 1.42	72.64 ± 2.07	72.06 ± 1.68	71.80 ± 0.87	71.23 ± 0.92
11	73.96 ± 1.42	61.98 ± 6.50	59.55 ± 5.21	63.71 ± 2.32	66.50 ±3.77

Table S3 – Data shown visually in Figures 4 and 5 in the manuscript

SAM Type	Carbon chain length	Thermogalvanic power / mW m^{-2}<br>Incubation Time	
		6 h	24 h
None	-	74.0 ± 1.4	74.0 ± 1.4
Cationic $[NMe_3]^+$	2	72.1 ± 1.7	71.2 ± 0.9
	5	--	67.8 ± 2.7
	11	59.6 ± 5.2	66.5 ± 3.8
Neutral OH	3	71.3 ± 0.4	71.7 ± 0.9
	6	-	59.7 ± 2.2
	8	-	26.7 ± 4.3
	11	12.4 ± 13.3	2.1 ± 1.2
Anionic $[COO]^-$	3	62.6 ± 3.9	69.2 ± 6.4
	6	-	23.0 ± 11.5
	11	14 ± 14	3.0 ± 0.9

SAM protection of Au against passivation – tabulated data of fitted Nyquist plots

Table S4 – Table of data for SAM protection of Au, as shown in Figure 6.

Investigated system	Treatment	R_S / Ω	R_{ET} / Ω
Clean Au	Freshly cleaned	38.0 ± 0.5	59.8 ± 1.2
	Soaked in $[Fe(CN)_6]^{3-/4-}$	34.31 ± 0.4	575.7 ± 2.5
Au with cationic C2 (+) SAM	SAM freshly prepared	31.5 ± 1.5*	<10
	Soaked in $[Fe(CN)_6]^{3-/4-}$	33.0 ± 0.7	39.4 ± 1.0
Au with cationic C5 (+) SAM	SAM freshly prepared	33.5 ± 0.3*	<10
	Soaked in $[Fe(CN)_6]^{3-/4-}$	32.1 ± 0.6	54.4 ± 1.1
Au with cationic C11 (+) SAM**	SAM freshly prepared	30.0 ± 0.2	557.1 ± 1.2

* This R_s was determined by fitting with a fixed R_{ET} of 10 Ω
** Since the cationic C11 SAM resulted in R_{ET} values consistent with $[Fe(CN)_6]$-passivated Au, exploring Au protection with this SAM was not investigated.

SAM protection of Au against passivation - Nyquist plots

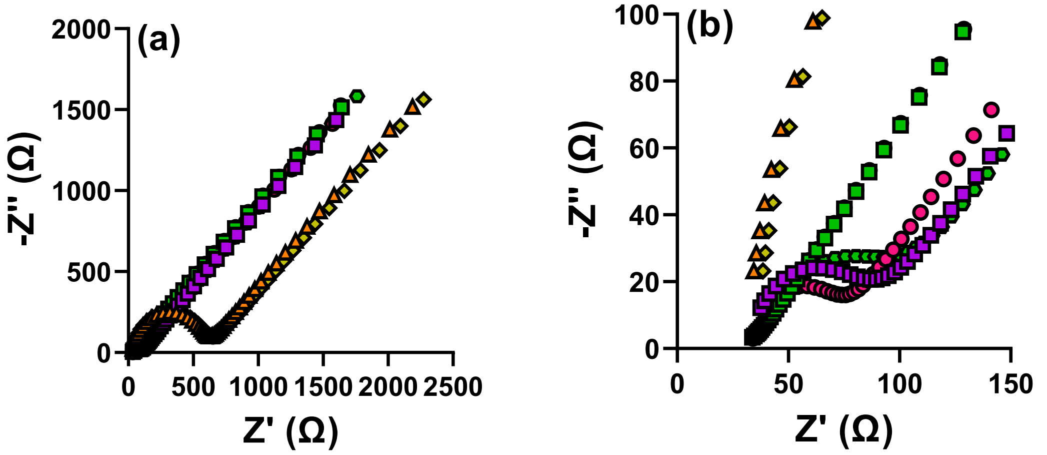

Figure S10 – Figure showing Nyquist plots of (green hexagon) clean Au, (yellow diamond) clean Au soaked in $[Fe(CN)_6]^{3-/4-}$, (blue circle) clean Au with C2 (+) SAM attached, (orange circle) C2 (+) SAM@Au soaked in $[Fe(CN)_6]^{3-/4-}$, (green square) clean Au with C5 (+) SAM attached, (purple square) C5 (+) SAM@Au soaked in $[Fe(CN)_6]^{3-/4-}$ and (red triangle) clean Au with C11 (+) SAM attached. Information on the frequency range used to fit the data is given in Figure S8.

SAM protection of Au against passivation – fitted Nyquist plots

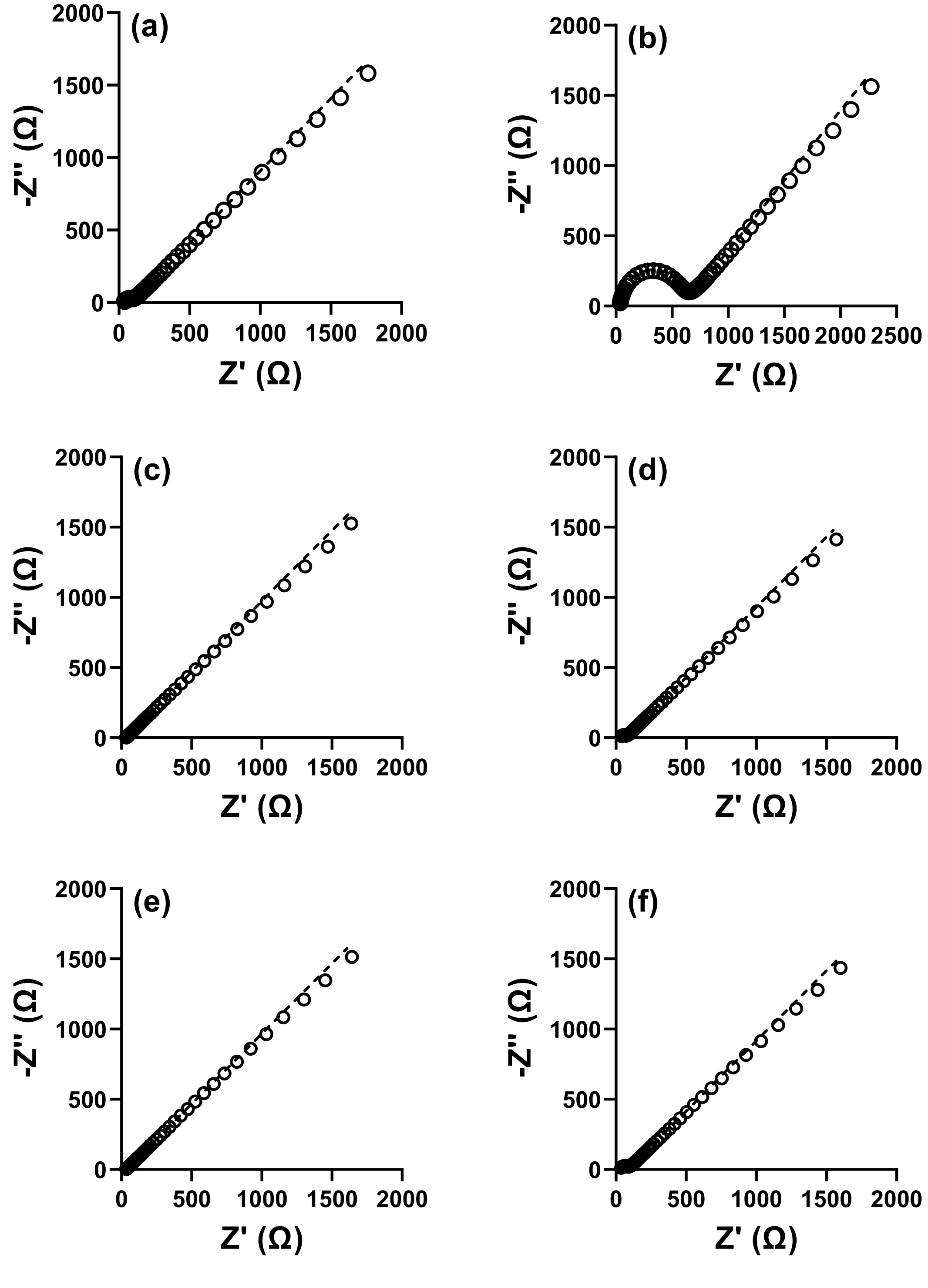

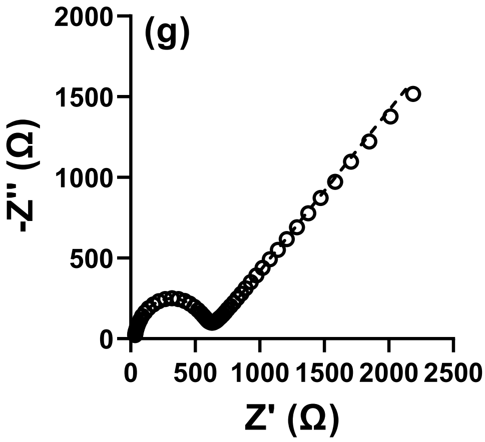

Figure S11 - Figure showing Nyquist plots of (a) clean Au (fit to the frequency range 50 kHz – 0.1 Hz), (b) clean Au soaked in $[Fe(CN)_6]^{3-/4-}$ (fit to the frequency range 50 kHz – 0.1 Hz), (c) clean Au with C2 (+) SAM attached (fit to the frequency range 50 kHz – 0.1 Hz), (d) C2 (+) SAM@Au soaked in $[Fe(CN)_6]^{3-/4-}$ (fit to the frequency range 50 kHz – 0.1 Hz), (e) clean Au with C5 (+) SAM attached (fit to the frequency range 50 kHz – 0.1 Hz), (f) C5 (+) SAM@Au soaked in $[Fe(CN)_6]^{3-/4-}$ (fit to the frequency range 50 kHz – 0.1 Hz) and (g) clean Au with C11 (+) SAM attached (fit to the frequency range 50 kHz – 0.1 Hz).

Electrocatalytic comparison of unmodified electrode materials (platinum, gold and glassy carbon)

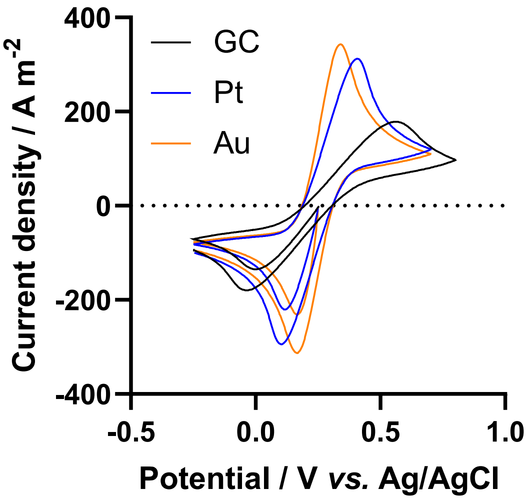

Figure S12 - Cyclic voltammetry of aqueous 0.2 M $K_3/K_4[Fe(CN)_6]$ (0.1 M each, with no other supporting electrolyte) recorded using gold (orange), platinum (blue) and glassy carbon (black) electrodes. The smaller peak-to-peak separation demonstrations gold is the most effective electrocatalytic metal for this concentrated $K_3/K_4[Fe(CN)_6]$ system.
